# KRAB-ZFPs and cancer stem cells identity

**DOI:** 10.1016/j.gendis.2022.03.013

**Published:** 2022-04-09

**Authors:** Anna Olechnowicz, Urszula Oleksiewicz, Marta Machnik

**Affiliations:** a Department of Cancer Immunology, Poznan University of Medical Sciences, Poznan 60-806, Poland; b Department of Histology and Embryology, Poznan University of Medical Sciences, Poznan 60-781, Poland; c Doctoral School, Poznan University of Medical Sciences, Poznan 60-812, Poland; d Department of Cancer Diagnostics and Immunology, Greater Poland Cancer Centre, Poznan 61-866, Poland

**Keywords:** Cancer, Cancer stem cell, Epigenetic, KRAB-ZFP, Transcription factors

## Abstract

Studies on carcinogenesis continue to provide new information about different disease-related processes. Among others, much research has focused on the involvement of cancer stem cells (CSCs) in tumor initiation and progression. Studying the similarities and differences between CSCs and physiological stem cells (SCs) allows for a better understanding of cancer biology. Recently, it was shown that stem cell identity is partially governed by the Krϋppel-associated box domain zinc finger proteins (KRAB-ZFPs), the biggest family of transcription regulators. Several KRAB-ZFP factors exert a known effect in tumor cells, acting as tumor suppressor genes (TSGs) or oncogenes, yet their role in CSCs is still poorly characterized. Here, we review recent studies regarding the influence of KRAB-ZFPs and their cofactor protein TRIM28 on CSCs phenotype, stemness features, migration and invasion potential, metastasis, and expression of parental markers.

## Introduction

Genetic changes and epigenetic processes in cancer cells may initiate and influence each other.^1^ Mutations within the genome, as well as multiple epigenetic changes, frequently result in augmented tumor heterogeneity. Indeed, in most cases, cancer contains various cell subpopulations, including cancer stem cells (CSCs). An increasing amount of research indicates that CSCs may be responsible for metastasis, resistance to therapies, and subsequently, worse patient prognosis.^2^^,^^3^

The function of many proteins is frequently perturbed in cancer cells,^4^^,^^5^ including Krϋppel-associated box domain zinc finger proteins (KRAB-ZFPs). KRAB-ZFPs belong to a large family of epigenetic repressors. Some of these factors play an essential role in stem cell biology and differentiation processes.^6^, ^7^, ^8^ Moreover, the pan-cancer transcriptomic analysis revealed that many KRAB-ZFPs are commonly downregulated or overexpressed in multiple tumor types.^9^^,^^10^ A growing amount of data indicate that some of them may function as oncogenes, while others as tumor suppressor genes (TSGs).^11^ Nevertheless, the specific role of the majority of KRAB-ZFP family members remains unexplored, and consequently, little is known about their functioning in CSCs. Combining the knowledge about individual factors and their functioning in physiological SCs like embryonic stem cells (ESCs), adult stem cells (ASCs) or hematopoietic stem cells (HSCs) and cancer cells may shed some light on CSCs biology. This issue needs to be better studied in order to reveal the potential role of KRAB-ZFPs in the maintenance of pluripotency and cancer aggressiveness. Physiological SCs and CSCs share many similarities; hence it is possible that KRAB-ZFPs will have a common effect on both cell types. Currently, many studies are focused on the improvement of anti-cancer therapies. As mentioned above, CSCs may be responsible for cancer recurrence, metastasis, and poor patient outcome, so it is crucial to characterize them in more detail. Below, we will focus on these observations to summarize the data indicating that KRAB-ZFPs may have an important role in CSCs.

## Cancer formation via interconnected genetic and epigenetic aberrancies

The knowledge regarding cancer origin, biology, and treatment options is still expanding, yet it remains the second leading cause of death.^12^ During oncogenesis accumulating genetic and epigenetic aberration lead to the acquisition of hallmark cancer features, including constant signaling of proliferation, avoidance of growth suppressors, replicative immortality, resistance to cell death, induction of tumor angiogenesis, invasiveness and metastasis, presence of mutations in the genome and its instability, inflammation that promotes tumor formation, avoidance of immune responses, and reprogrammed cellular metabolism.^13^^,^^14^ All of these features influence the course of carcinogenesis, drug susceptibility, and patient life expectancy.

A wide array of driver mutations were identified to date across multiple tumor types through classical methods or next-generation sequencing.^15^, ^16^, ^17^ Mutations may affect the DNA sequence of a single gene or non-coding RNA (ncRNA), as well as a chromosome structure and copy number. These mutations may be inherited or stimulated by a variety of external and internal factors, e.g., smoking, viral infections, inflammation, irradiation, etc. As mentioned above, apart from the genetic and genomic alterations, cancer formation may be driven by disturbed epigenetic signaling.^4^ The epigenomic profile of a given cell drives its transcriptional signature. Thus, the cells with the same genotype but different epigenomes are able to perform distinct transcriptional programs, and as such – exhibit divergent functions.^18^^,^^19^ In certain cases, epigenetic changes may affect cancer cell phenotype more profoundly than genetic mutations, as may be observed in several childhood malignancies, including retinoblastoma^20^ or medulloblastoma.^21^^,^^22^ Nevertheless, in the majority of cancers, genetic mutations and epigenetic aberrations (so-called epimutations) may coincide and influence each other.

One of the best-characterized epigenetic events is cytosine methylation within CpG dinucleotides. Cancer cells frequently harbor a specific pattern of DNA methylation: genome-wide hypomethylation accompanied by local CpG hypermethylation.^4^^,^^23^ Hypermethylation in cancer frequently occurs in the promoters of TSGs and DNA repair genes and may lead to a decline in their expression.^24^ In contrast, global DNA hypomethylation may disturb chromatin structure and epigenetic regulation. Moreover, it may contribute to the augmented genomic instability and activation of oncogene transcription.^4^^,^^25^ Besides changes within DNA methylation signature, cancer cells harbor numerous epigenetic alterations related to histone post-translational modifications (PTMs), ncRNA expression profile, and 3D chromatin organization. As could be expected, many chromatin modifiers exhibit aberrant functioning in cancer due to mutations or changes in their expression level.^5^^,^^26^^,^^27^ In many cancer types, the chromatin factors responsible for the maintenance of heterochromatin are overexpressed, while those involved in chromatin relaxation are downregulated or mutated.^4^^,^^5^ Therefore, PTMs may become largely re-distributed in tumors compared to their normal counterparts,^28^^,^^29^ as well as between cancer cells at various differentiation states, e.g., metastatic *vs*. non-metastatic cells.^30^ These global alterations rewire the transcriptional program that supports the maintenance or switch between distinct cancerous phenotypes.^30^, ^31^, ^32^ For instance, H3K9me2 is a repressive mark that accumulates during cell differentiation within transcriptionally silenced sites. These regions are significantly lost in cancer cells, which likely contribute to enhanced phenotypic plasticity observed as well in ESCs.^33^ Also, H3K9me3 levels may drop during carcinogenic transformation as demonstrated in the case of oncogene promoters, pericentromeric heterochromatin, and transposable elements (TEs).^29^^,^^34^, ^35^, ^36^ Such an event may lead to genomic instability via inappropriate chromosome segregation,^35^ as well as TE-mediated insertional mutagenesis predisposing to recombination.^36^ Interestingly, the upregulation of H3K9 methyltransferases: SET domain bifurcated 1/2 (SETDB1/2) and subsequent increase in H3K9me3 deposition are frequently linked to the acquired drug resistance in many tumor types.^32^^,^^37^^,^^38^ A study by Al Emran and colleagues focused on histone modifications in induced drug tolerance in cancer cells (IDTCs) .^37^ By examining melanoma cell lines with IDTCs, it was shown that SETDB1 levels were higher in cells with drug resistance, thereby increasing the presence of H3K9me3, possibly affecting gene repression in IDTCs.

Epigenetic alterations occurring in cancer cells may increase cellular plasticity in a growing tumor.^4^ The available data indicate that tumor mass may be highly heterogeneous, and the composition of distinct cell populations may vary during the course of the disease.^39^, ^40^, ^41^, ^42^ Such changes in heterogeneity within the tumor may be related to the presence of cells with stem cell-like features known as CSCs.

## Stem cells and cancer stem cells

CSCs make up a small part of the tumor mass and may be identified at various cancer stages.^3^^,^^43^ They are slowly growing, self-renewing cells capable of withstanding harsh conditions, such as hypoxia, nutrient deprivation, or anti-cancer drug administration. Furthermore, CSCs may undergo epithelial-to-mesenchymal transition (EMT) that facilitates mobility and metastasis.^44^, ^45^, ^46^ Such plasticity of CSCs may be ascribed to epigenetic events that enable the fast acquisition of traits supporting adaptation to changes within the cancer environment.

Embryonic SCs (ESCs) have the ability to differentiate into virtually any cell type.^47^ Also, CSCs produce various classes of neoplastic cells.^3^^,^^48^^,^^49^ Nevertheless, the origin of CSCs is difficult to trace. Hypothetically, they may appear as a consequence of perturbed balance between self-renewal and differentiation of physiological stem cells. Alternatively, they may arise upon the induction of *de novo* self-renewal capacity within somatic cells. Due to their unique features, CSCs may initiate tumor growth and maintain cell division.^47^^,^^50^ However, not every cell has such a founder cell potential.^43^

After the first discovery of CSCs in AML, they were identified in many types of tumors, including breast,^51^ lung,^52^ and brain cancers.^53^ A subset of cells with stemness properties may be isolated from the tumor mass based on the presence of specific surface markers. In breast cancer, they are detected by CD44^+^/CD24^−/low^ phenotype.^51^ Isolated breast cancer cells with CD44^+^/CD24^−^ profile are able to form mammospheres *in vitro* and xenografts in mice model *in vivo*. Additionally, these cells express the pluripotency-related gene *OCT4*.^54^ Of note, in many cases, the identification of CSCs within the tumor of particular origin is based on the gene expression signature that is also characteristic to normal stem cells.

Indeed, physiological SCs and CSCs share many common characteristics, although several essential differences can be noted (Table 1). They demonstrate high telomerase activity, resistance to DNA damage, and the ability to self-renew and differentiate into more specialized cell lineages.^47^^,^^55^ However, in CSC, the differentiation process may be disturbed, and the emerging cells may be incorrect.^47^ Instead of engaging proper differentiation signaling pathways, CSCs utilize some of the pathways active in normal stem cells. An example is the JAK/STAT pathway, where STAT3 protein is up-regulated in a population of stem-like breast cancer cells in comparison to non-stem-like breast cancer cells.^56^ STAT3 is known for its positive effect on maintaining the undifferentiated state in mouse ESCs (mESCs)^57^ and for promoting hMSCs (human mesenchymal stem cells) migration to breast cancer cells.^58^ This may suggest that upregulation of STAT3 in CSCs is necessary for its stem-like nature.

**Table 1:** Comparison of physiological SCs, CSCs and regular cancer cells. All types of cells possess cell-specific characteristics but also share some common features.

Features	Physiological stem cells	Cancer stem cells	Regular cancer cells
Self-renewal ability	Enables tissues to be built from progenitor cells^59^	Enables production of cancer cells that form a tumor mass^59^	No self-renewal capability
Telomerase	Active	Active	Active in about 85% of all tumor types^60^
Pluripotency markers	OCT4, NANOG, SOX2,^61^ SALL4,^62^ STAT3,^63^ c-Myc,^64^ Klf4^65^	OCT4, NANOG, SOX2,^66^ SALL4,^67^ STAT3,^68^ c-Myc,^64^ Klf4^69^	SALL4,^70^ STAT3,^71^ c-Myc^72^
Susceptibility to chemotherapy	Resistant	Resistant	Sensitive according to tumor type
Markers	CD29^high/+^,^73^^,^^74^ CD34^+^,^75^^,^^76^ CD44^+^,^74^ CD49f^high/+^,^73^, ^74^, ^75^, ^76^, ^77^ CD90^+^,^74^^,^^76^ CD117^+^,^76^ CD133^+^,^73^ CD184^+^,^76^ CD200^+^,^77^ Sca1^+^^74^^,^^77^	Annexin II^+^ (AII^+^),^78^ ALDH^high^,^78^ CD34^low/high^,^79^ CD44^+^/CD24^−/low^,^51^ CD49f^+^,^78^^,^^82^, ^81^, ^80^ CD133^+^,^82^^,^^83^ CK-17,^78^ p63^+^^78^	CCR10^high^,^84^ CD9,^85^ CD63,^86^ CD82,^87^ CD151,^88^ CD182^low^,^84^ CD184^high/+^,^84^^,^^89^ CD197^high^^84^
Differentiation	Differentiate to almost every type of cell; Possibility to change into CSC	Change into a cancer cell	Already differentiated cells
Function of cells in relation to their place of origin	Replacing old cells in the body; Creating different germ layers	Forming metastasis	Forming tumor mass
Signaling pathways	Signal pathways functioning properly	Altered in comparison to the regular functioning of pathways JAK-STAT, Hedgehog pathway – overexpression of involved genes WNT – activation of the pathway PI3K – repressed by miR-126^90^	Altered pathway genes according to tumor type; The most commonly altered pathways – RTK/RAS/MAP-Kinase (46% of examined cancer samples), cell cycle (45%), PI3K (33%), TP53 (29%), NOTCH (23%), WNT (15%), MYC (11%), HIPPO (10%), TGF-Beta (7%) and NRF2 (1%)^91^

As mentioned above, CSCs may be recognized by the biomarkers that are also characteristic for physiological stem cells. For example, cell surface markers, such as CD133, may be found in both CSCs and regular SCs like HSCs or ASCs.^47^^,^^92^^,^^93^ CD133 detected on CSCs in glioblastoma, pancreatic adenocarcinoma, hepatocellular carcinoma (HCC), and brain tumor,^50^^,^^94^, ^95^, ^96^ is also a marker of HSCs and neural stem cells (NSCs).^50^^,^^97^ Research conducted by Ma and colleagues showed that CD133^+^ HCC cells also had enhanced expression of various stemness factors (e.g., β-catenin, OCT3/4, iBMI, SMO, and NOTCH1).^95^ In glioblastoma, CD133 positive cancer cells exhibit resistance to chemotherapy.^98^ Also, ALDH1 shows high expression in physiological SCs, and in CSCs in various tissues. Augmented ALDH1 levels in breast, bladder, and prostate cancers correlate with poor prognosis.^99^, ^100^, ^101^ In thyroid neoplasm, ALDH^High^ CSCs show resistance to chemotherapy due to the activation of Hedgehog pathway.^102^ Cells with high ALDH levels are also characterized by increased expression of stemness genes, i.e., OCT4, NANOG, and SOX2.^103^

It is well established that CSCs are responsible for tumor migration and metastasis.^3^ Thus, they were divided into two subtypes responsible for different functions: primary CSCs (pCSCs), which form the tumor and exist in its environment, and migrating CSCs (mCSCs), which are responsible for metastasis.^50^ Studies conducted on breast CD44^+^/CD24^−^ CSCs showed that OCT4 and NANOG have a significant impact on metastasis. Silencing both factors simultaneously reduced the expression of genes that positively affect EMT. The opposite effect was noted in the case of overexpression – the levels of EMT-related genes were increased. Also, the same relationship occurred in *in vitro* culture of CSCs in terms of cell migration.^104^ Similar results were observed in lung adenocarcinoma cell line A549.^105^ A study by Xu and colleagues on colorectal cancer also showed that higher NANOG-expression correlated with more frequent postoperative liver metastases.^106^ Furthermore, Lu and colleagues showed that Nanog induced migratory, invasive, and metastatic potential of mammary cancer cells in a mouse model.^107^ They observed metastases to the lungs, liver, and kidneys. Notably, the cells with high Nanog expression were characterized by an increased expression of Pdgfrα, which positively affects carcinogenesis and metastasis.^107^ Nevertheless, stem cell markers may also show a negative impact on metastasis. For example, the study of Shen and colleagues in *in vitro* and *in vivo* mouse models showed that the overexpression of Oct4 reduced the metastatic potential of breast cancer cells. This effect was ascribed to the findings that Oct4 reduces the expression of Rnd1, which is responsible for cell adhesion.^108^ Such opposing observations may be related to the differences in utilized research models, and particularly to their genomic and epigenomic make-up. Clearly, further studies are needed to improve our understanding of SC markers' involvement in cancer biology.

## KRAB-ZFPs

The aforementioned OCT4 and NANOG are well-known factors regulating pluripotency-related features. The knowledge about other proteins affecting physiological and cancer stem cells is yet still expanding. One example is TRIM28 (tripartite motif protein 28), also known as KRAB-associated protein 1 (KAP1). It interacts and co-operates with KRAB-ZFP factors in mediating epigenetic repression. TRIM28 and several KRAB-ZFP factors were shown to participate in the maintenance of pluripotency in human embryonic stem cells (hESCs).^7^ As mentioned earlier, the expression of many KRAB-ZFPs is deregulated in multiple tumor types,^9^^,^^10^ and accumulating evidence suggests that some of the KRAB-ZFP factors play a role in oncogenesis.^11^ Based on the connection between KRAB-ZFP, stemness, and cancer, we will review the available literature that points towards the potential involvement of TRIM28 and KRAB-ZFPs in CSCs phenotype. KRAB-ZFPs belong to the relatively young family of transcriptional repressors containing a KRAB domain and a variable number of zinc finger motifs. They emerged around 420 million years ago in a common ancestor of coelacanths lungfish and tetrapods.^109^ The human genome comprises approximately 381 KRAB-ZFP genes, which may form over 800 transcripts.^110^ KRAB-ZFPs play an essential role in the transcriptional regulation of various signaling pathways, thus affecting many biological processes, including apoptosis, cell cycle, development, and stem cell differentiation.^7^^,^^11^^,^^111^^,^^112^ The KRAB domain may be composed of two parts: A-box, which is necessary for the repressive function of the protein, and B-box, which enhances the repressive effect.^6^^,^^113^ KRAB-ZFPs may also contain additional domains, such as SRE-ZBP, CTfin51, AW-1, and Number18 cDNA domain (SCAN) or Domain of Unknown Function 3669 (DUF3669).^6^

The canonical function of KRAB-ZFPs is epigenetic suppression through the deposition of repressive chromatin marks. In addition, KRAB-ZFPs may also facilitate DNA methylation within the affected locus. However, this effect seems to occur only in the cells with stem cell phenotype.^7^^,^^8^ KRAB-ZFPs bind DNA via zinc finger motifs, whereas the KRAB domain recruits its corepressor – TRIM28. The KRAB domain interacts with the Ring finger/B box/Coiled-Coil (RBCC) domain at the N-terminus of TRIM28.^111^^,^^112^^,^^114^^,^^115^ The C-terminal domain and bromodomain recruit Nucleosome Remodelling Deacetylase (NuRD) complex and histone lysine methyltransferase SET Domain Bifurcated 1 (SETDB1), thus mediating histone deacetylation and H3K9me3 deposition, respectively. The central part of TRIM28 binds to Heterochromatin Protein 1 (HP1), which recognizes H3K9me3 and promotes regional heterochromatization.^112^^,^^116^^,^^117^ The repressive functions conferred by KRAB-ZFPs may affect gene promoters and 3′ ends, imprinted control regions, and various classes of TEs.^109^

An increasing number of published reports indicate that KRAB-ZFP may play an important role in cancer, affecting cancer cell phenotype, as well as disease prognosis and patient survival (reviewed in^11^). The data from various tumor tissues and experimental models show that KRAB-ZFPs may act both as TSGs (e.g., *ZNF23*, *ZNF382*, *ZNF471*, ZNF545)^118^, ^119^, ^120^, ^121^ or oncogenes (e.g., *ZNF300*, *ZKSCAN3*).^122^^,^^123^ Their imbalanced expression results in the aberrant stimulation of various cancer-related signaling pathways (e.g., p53, NFkB, and Wnt/β-catenin).^11^ An interesting example of oncogenic transcriptional repression over several regions was demonstrated in the case of ZNF304 in colorectal cancer.^124^ *ZNF304*, whose expression rises upon mutant KRAS signaling, was shown to mediate the coordinated silencing of several loci, thus contributing to the CpG island methylator phenotype. In the study by Serra and colleagues, ZNF304 attached to the INK4-ARF locus that encodes *p14*^*ARF*^, *p15*^*INK4B*^, and *p16*^*INK4A*^ TSGs, and recruited other epigenetic corepressors, including TRIM28, SETDB1, and DNMT1.^124^ Of note, the published evidence suggests that KRAB-ZFPs may act not only as gene co-suppressors but also as co-activators depending on the protein partners interacting with a given KRAB-ZFP factor.^11^^,^^125^, ^126^, ^127^

## KRAB-ZFP/TRIM28 influence on stem cells and cancer stem cells

### TRIM28

**Figure 1:**
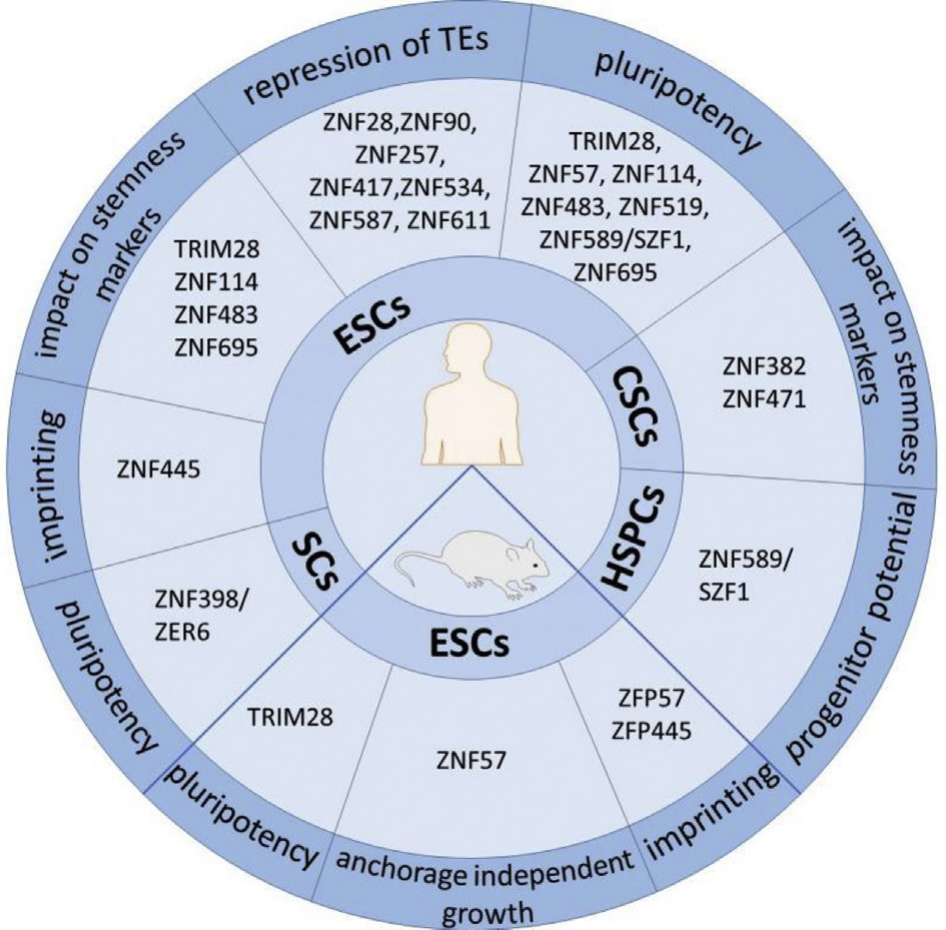
TRIM28 and KRAB-ZFPs are involved in different biological processes in physiological and cancer stem cells.

KRAB-ZFP cofactor protein, TRIM28, influences both stem cells and cancer stem cells (Fig. 1).^114^^,^^128^ Its essential role in the functioning of SCs was demonstrated in the research carried out by Hu and colleagues in the mESCs.^129^ Lack of Trim28 triggered ES differentiation into the primitive ectoderm lineage. Interestingly, Trim28-targeted transcription regulators of the cell cycle, cell death, and cancer, supporting the idea that self-renewal-specific pathways may also be active in certain cancers.^129^ In another study, Seki and colleagues demonstrated that induced overexpression of Trim28 sustained the pluripotency of mESCs. In contrast, Trim28 silencing resulted in a decreased growth, change in ESCs morphology (cells became stretched and less tightly compacted), and reduced expression of pluripotency markers, namely SSEA1 and Nanog.^128^ It is worth mentioning that Trim28 is indispensable for early development and mouse embryos with *Trim28* knockout are lethal.^130^^,^^131^ Therefore, all changes observed in ESCs upon Trim28 knock-down should be analyzed carefully to confirm that they are not a side effect in dying cells.

In hESCs, TRIM28 silencing triggered differentiation manifested with morphological changes as well as the reduction of expression of pluripotency markers: NANOG, OCT3/4, and TRA-1-60.^7^ Similar to physiological SCs, TRIM28 affects stem cell features in the cancer setting. The study conducted by Czerwińska and colleagues^114^ revealed increased expression of TRIM28 in 14 tumor types from The Cancer Genome Atlas (TCGA) project, e.g., lung, breast, gastric, and pancreatic cancer. Moreover, a high TRIM28 level was associated with the patient's outcome. For example, in gastric cancer, high TRIM28 expression correlated with lower survival rate.^114^ Conversely, in the early stage of lung cancer, high level of TRIM28 expression correlated with better overall patient survival.^132^ Knock-down of TRIM28 *in vitro* had no major effect on proliferation and stemness in breast cancer cell lines. However, in *in vivo* experiments, a lower TRIM28 level was shown to be related to weaker self-renewal and tumor formation properties. Xenografts obtained from the TRIM28^KD^ MDA-MB-231 breast cancer cell line (that contains an enriched CD44^+^/CD24^−/low^ subpopulation) exhibited reduced tumor formation. This effect was not observed in the TRIM28^KD^ MCF7 cell line with observed low content CD44^+^/CD24^−/low^ cells. Molecular profiling revealed that TRIM28 silencing was associated with lower expression of stem cell markers, including OCT3/4, SOX2, and NANOG, and with the metabolic switch from oxphos to glycolysis.^114^ Altogether, these data indicate that TRIM28 participates in the stemness maintenance in both physiological and cancer stem cells.

### KRAB-ZFPs

**Figure 2:**
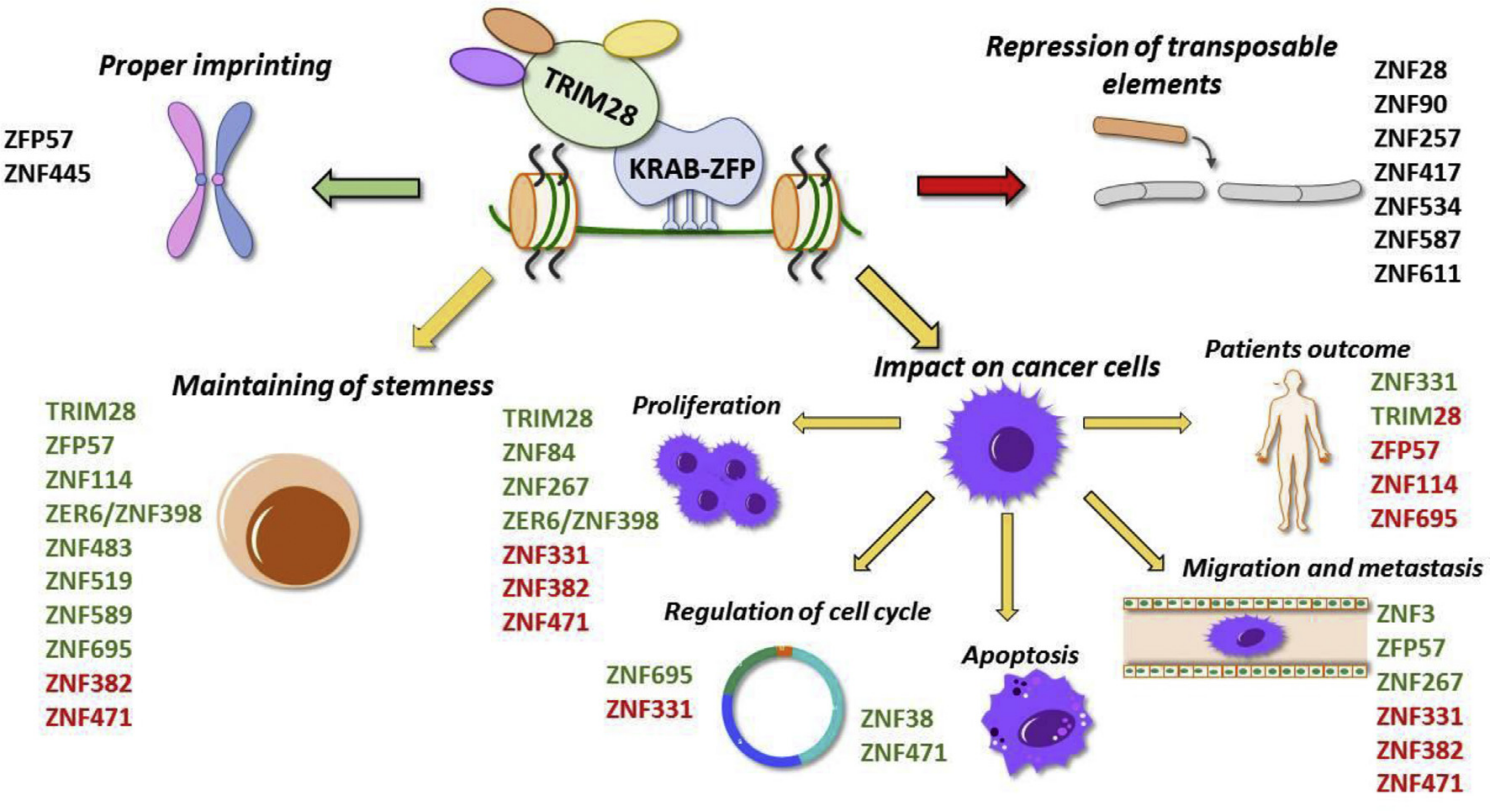
KRAB-ZFP/TRIM28 protein complex is involved in activation (green) and repression (red) of several biological processes.

As mentioned above, KRAB-ZFP factors take part in the functioning of differentiated and undifferentiated cells (Fig. 2).^7^ In our previous research, we used transcriptomic data from home-derived dermal fibroblasts and iPSCs, as well as the Progenitor Cell Biology Consortium (PCBC), focused on the iPS induction and differentiation to various cell lineages.^133^ In both datasets, we identified 38 KRAB-ZFPs up- and down-regulated in pluripotent stem cells compared to their more differentiated counterparts. We confirmed that at least 10 of them are up-regulated in iPSCs compared to fibroblasts. Furthermore, their level increased during somatic cell reprogramming to iPSC and vice versa – their level decreased with iPSCs differentiation into somatic cells. To determine the functional effect of certain KRAB-ZFPs on pluripotency, ZFP57, ZNF114, ZNF483, ZNF519, ZNF589, and ZNF695, as well as TRIM28, were knocked down in hESCs. The silencing caused changes in cell morphology – cell elongation, dispersion, and loss of colony boundaries, which suggested cell differentiation. Besides, silencing TRIM28, ZNF483 and ZNF695 was followed by a decrease in NANOG and OCT3/4 levels. The data showed as well that KRAB-ZFPs may target the promoters of genes implicated in developmental biology, cell cycle, and extracellular matrix organization. Moreover, during reprogramming of dermal fibroblasts to iPSCs, KRAB/TRIM28 axis mediated promoter silencing via deposition of H3K9me3 and DNA methylation.^7^

Another function important for stemness that occurs during embryonic development is the ability of KRAB-ZFP factors to restrict various classes of transposable elements. For example, ZNF90, ZNF257, and ZNF534 repress human endogenous retrovirus K (HERVK), while ZNF28 and ZNF611 silence SINE-VNTR-Alu (SVA) during embryonic genome activation (EGA). These factors target recent TEs to rewire transcriptional programs in naive hESCs.^134^ Other KRAB-ZFPs associated with ESCs and TEs are ZNF417 and ZNF587. These factors repress HERVK and SVA during EGA in ESCs, which later differentiate into neurons.^135^ TEs take part in the regulation of pluripotency – a lot of binding sites intended for pluripotency factors are found within endogenous retroviruses (ERVs).^6^ Almost one-fifth of the Oct4–Sox2 binding sites are located in mouse endogenous retrovirus K (ERVK) and may therefore be affected by KRAB-ZFP.^136^ Moreover, ZFP57 and ZNF445/ZFP445 are involved in the control of gene imprinting.^137^ In mESCs, both factors are responsible for the proper imprinting pattern within imprinting control regions (ICRs). However, in hESCs, ZNF445 has greater importance in the regulation of imprinting.^137^

Transcriptomic profiling of different cell populations within the tumor mass demonstrate that distinct members of the KRAB-ZFP family are found among genes specifically up- or downregulated in CSCs. Seo and colleagues detected 49 genes that were significantly downregulated and 12 up-regulated in a lung adenocarcinoma cell line subpopulation with stem cell properties. Two KRAB-ZFP transcription factors, namely ZNF567 and ZNF267, were among genes showing higher expression in CSCs.^138^ The function of ZNF567 is yet unknown, whereas ZNF267 is up-regulated in liver cirrhosis^139^ and HCC cell lines and tissues^140^ where it positively correlates with proliferation and migration. ZNF267 might be useful as a prognostic biomarker of HCC development since its expression is also elevated in pre-cancerous tissues.^140^

Bhat-Nakshatri and colleagues identified a perturbed expression pattern of several KRAB-ZFPs in CSCs subpopulation of the MCF10A cell line. ZNF84, ZNF606, ZNF585A, ZNF420, ZNF41, ZNF25, ZNF226, ZNF222, and ZNF136 were up-regulated, whereas ZNF552 was downregulated in stem cell-like cells.^141^ Possibly, ZNF84 may function as an oncogene since its expression is elevated in cervical cancer tissues and positively correlates with tumor size and cell proliferation.^142^ In contrast, ZNF606 might act as a TSG. Its expression is downregulated via promoter hypermethylation in colorectal cancer with mutated *BRAF*.^143^ Recently, the CSCs subpopulation of another two breast cancer cell lines was characterized.^144^^,^^145^ CSCs isolated from the basal breast cancer cell line MDA-MB-468 were subjected to whole-transcriptome analysis in order to define stem cell-related gene expression signature. Only one KRAB-ZFP factor, ZNF354A, was significantly downregulated in the CSC population.^145^ The same factor was detected in our previous studies analyzing KRAB-ZFPs expression in the TCGA datasets.^10^ The level of ZNF354A was decreased in BRCA, as well as in several other types of cancer samples, i.e., thyroid, prostate, and kidney. Interestingly, the same factor was elevated in other analyzed types, like lung adenocarcinoma, liver cancer, or cholangiocarcinoma.^10^ The CSC transcriptomic profiling of the MDA-MB-231 BRCA cell line utilizing a single-cell sequencing approach identified only one differentially expressed KRAB-ZFP, namely ZNF566.^144^ Its expression was found downregulated in CSC-like cells compared to non-CSCs. However, its role in cancer remains unknown.

A single-cell approach demonstrated stem-cell-related features of subpopulations within glioblastoma^146^ and leukemia cells.^147^ In the case of patients with chronic myeloid leukemia, only one KRAB-ZFP was detected among stem cell-related genes (ZNF302). The expression of ZNF302 was decreased in CSCs in comparison to normal HSCs, but its role was not explored further in the cancer setting.^147^ Furthermore, Zhao and colleagues analyzed transcriptomes of two neural cell lines: a glioblastoma CSC line and a neural stem cell line (NSCs). Three KRAB-ZFPs (ZNF92, ZNF480, and ZNF3) were found within genes with increased expression that distinguished CSCs from NSCs.^146^ ZNF92 was previously found to be highly expressed in multiple cancers, i.e., breast, lung, liver, and bladder.^10^ ZNF3 is one of the genes that were proposed in a multigene predictor set of metastatic outcome in BRCA patients^148^ and as an epigenetic marker for gastric cancer risk development in patients with *H. pylori* infection.^149^ The function of ZNF480 in carcinogenesis remains unknown.

The role of individual KRAB-ZFP factors in CSCs remains poorly characterized, although certain aspects of their potential engagement in CSCs biology may be pinpointed as reviewed below. The known function of specific KRAB-ZFPs in both cancer and embryonic cells is summarized in Table 2 and Figure 1.

**Table 2:** The function of specific KRAB-ZFPs in both cancer and embryonic cells.

KRAB-ZFP factor/TRIM28	Function in cancer cells	Function in stem cells	References
TRIM28	impact on lower survival rate in gastric cancer; positive effect on tumor formation	maintenance of pluripotency, self-renewal, and *OCT3/4* and *NANOG* gene expression	^7^^,^^114^^,^^128^^,^^129^
ZFP57	positive impact of overexpression on metastasis; positive association with NANOG expression; negative impact on patient outcome	maintenance of pluripotency; proper imprinting pattern within ICRs; influence on anchorage-independent growth	^7^^,^^137^^,^^150^, ^151^, ^152^
ZNF114	impact on lower cell survival	maintenance of pluripotency and stemness; repression of differentiation gene *DPYSL4*	^7^^,^^153^
ZNF331	inhibition of cell migration and invasion; inhibition of *c-Myc* and proliferation genes – *DDX5*, *EIF5A*, *GARS*, *STAM*, *UQCRFS1*, and *SET*	possible influence on breast CSCs by negative regulation of *c-Myc* expression	154, 155, 156
ZNF382	negative impact on EMT and metastasis	negative impact on *OCT4*, *SOX2*, and *NANOG* expression in CSCs	157, 158, 159
ZNF398/ZER6	positive impact on proliferation; impact on p53 ubiquitination	maintenance of pluripotency and inhibition of cell differentiation; influence on expression of TGF-β targeted genes	^160^^,^^161^
ZNF471	reduction of cell viability, migration and invasion; induction of apoptosis; inhibition of AKT and Wnt/β-catenin pathways	reduction of *CD44*, *NANOG2*, *OCT4*, *SOX2*, and *KLF4* expression in CSCs	^120^^,^^162^
ZNF695	upregulation of cell cycle genes	maintenance of pluripotency, and*OCT3/4* and *NANOG* gene expression in human ESCs	^7^^,^^163^

### ZNF695

ZNF695, tested for the effect on stemness in hESCs as described above,^7^ also seems to affect CSCs. For example, Li and colleagues focused on the master regulator genes in breast cancer that together may form a biomarker panel for the identification of specific molecular tumor subtype.^163^ Using three cohorts of human breast cancer data, they showed that one of the master regulator genes is *ZNF695*. Its expression differs depending on the tumor subtype – it is high in ER-negative subtypes (HER2-enriched and basal-like) and low in ER-positive (luminal tumors). Interestingly, ZNF695 was found to be associated with the upregulation of genes involved in cell cycle in basal-like and HER2-enriched subtypes. Also, ZNF695 overexpression was linked to mutations in the *TP53* gene, which frequently appear in ER-negative neoplasms.^163^ These features indicate greater aggressiveness of ER-negative neoplasms, especially in patients' survival from the first metastases. The differences between ER-negative and ER-positive tumors were described in the study by Kennecke and colleagues.^2^ ER-negative neoplasms (HER-2 enriched and basal-like subtypes) are less common than the luminal A subtype but are characterized by a lower survival rate. ER-negative tumors recur faster than luminal tumors, and median survival from the first distant metastasis is also lower.^2^ Noteworthy, the marker CD133, which is detected in many types of CSCs, has greater expression in ER-negative neoplasms than in ER-positive ones.^164^ Moreover, the CD44^+^/CD24^−^ phenotype is most common in basal-like breast cancer, which may indicate that this type of cancer has a higher percentage of CSCs.^165^ Considering the increased expression of ZNF695 in ER-negative tumors, it is tempting to speculate that its role in the cell cycle upregulation may be related to the aggressiveness of these tumor subtypes.

### ZFP57

*ZFP57*, a gene involved in imprinting maintenance, is positively regulated by NANOG. In both mESCs and human cancer cells, overexpression of NANOG resulted in increased expression of ZFP57, while its silencing caused ZFP57 down-regulation. Zfp57 was also demonstrated to influence the anchorage-independent growth of mESCs – knock-down of Zfp57 with simultaneous Nanog overexpression resulted in a reduction in the number of cell colonies.^152^ Higher expression of ZFP57 is thus associated with a greater proliferation potential of ESCs. Moreover, an increased expression of ZFP57 was also observed in grade IV compared to grade II glioblastoma.^150^ High-grade glioblastomas contain a higher content of CSCs, as evidenced by positive CD133 marker staining in over 60% of grade IV glioblastomas.^166^ Furthermore, ZFP57 overexpression resulted in higher metastatic potential in the mouse model of colorectal cancer, as reported by Shoji and colleagues.^151^ In the clinical samples, high levels of ZFP57 were shown to be positively associated with NANOG expression, as well as nodal and liver metastasis in colorectal cancer.^151^ Indeed, Nanog may participate in the upregulation of Zfp57 as demonstrated in mESCs.^152^ Moreover, ZFP57 upregulation correlated with poorer patient outcome.^151^ Altogether, these observations suggest ZFP57 may play a role in the maintenance and proliferation of CSCs.

### ZER6/ZNF398

ZER6/ZNF398 exists in two isoforms: p52-ZER6, which has a shorter version of the KRAB domain (truncated KRAB), and p71-ZER6 with an additional HUB-1 domain.^160^^,^^167^ ZER6 may act as a positive factor for the development of tumors, as shown in colorectal^160^ and breast cancers.^168^ In colorectal cancer, the p52-ZER6 isoform positively influences the interaction of p53 with MDM2, which leads to the ubiquitination of p53, and lowered expression of its target genes participating in cell cycle restriction. As could be expected, p52-ZER6 silencing resulted in increased p21 expression and inhibited the formation of xenografted tumors, indicating a positive effect of p52-ZER6 on the proliferation of cancer cells.^160^ The p71-ZER6 isoform, despite having a complete KRAB domain, cannot influence the interaction of p53 with MDM2 due to the presence of HUB-1 domain in its structure.^160^ Breast cancer cells positive for estrogen receptor alpha (ERα) also express both isoforms of the factor.^168^ p52-ZER6 interacts with ERα in the presence of 17β-estradiol, while p71-ZER6 through the aforementioned HUB-1 domain does not.^167^ From the molecular perspective, ZER6/ZNF398 was shown to promote the expression of genes that are targets for transforming growth factor β (TGF-β), which is mediated by colocalization with SMAD3 and histone acetyltransferase EP300.^161^ Owing to its role in cancer and in the regulation of stemness features, the influence of ZER6/ZNF398 on the phenotype of CSCs would be an interesting research direction. Noteworthy, ZER6/ZNF398 affects human pluripotent SCs by maintaining pluripotency and inhibiting cell differentiation. The factor is also required for the induction of pluripotency in fibroblasts.

### ZNF114

ZNF114 is another factor that contributes to the maintenance of pluripotency. Its expression was shown to become up-regulated during iPSC generation, whereas its knock-down caused a slight loss of stemness markers in hESCs. Additionally, ZNF114 repressed the *DPYSL4* gene involved in differentiation.^7^ It also affects the outcome in patients with clear cell renal cell carcinoma, as its higher expression was associated with shorter overall survival.^153^ Noteworthy, in gastric cancer cell line AZ-521, the silencing of pluripotency factor, SOX2, resulted in the upregulation of ZNF114.^169^ These observations may indicate that ZNF114 have a different functions in pluripotency-related processes depending on the cell type.

### ZNF471

Several KRAB-ZFP factors have a negative effect on the functioning of CSCs. An example is *ZNF471*, which is a TSG methylated in esophageal squamous cell carcinoma (ESCC)^120^ and breast cancer cells.^162^ Tao and colleagues investigated the effects of ZNF471 overexpression in MDA-MB-231 and YCC-B1 cell lines, demonstrating reduced cell viability, induction of apoptosis, and reduction of invasion and migration of cancer cells.^162^ Analogously, knock-down of ZNF471 increased cell growth, inhibited apoptosis, and promoted metastasis. These aspects may be related to the observed inhibitory effects of ZNF471 on AKT and Wnt/β-catenin pathways that influence tumor growth. In the case of the CSC subpopulation of tested cell lines, cells with higher ZNF471 levels had reduced expression of stemness markers, e.g., CD44, NANOG2, OCT4, SOX2, and KLF4.^162^ Thus, the inhibition of ZNF471 positively influences the development and growth of CSCs.

### ZNF382

ZNF382 also has a suppressor function against CSCs. This factor is expressed during embryogenesis mainly in the kidney and brain, while in adult cells, it is restricted to the heart tissue.^170^ It acts as a TSG – the expression is repressed by promoter methylation in esophageal cancer,^159^ gastric cancer,^158^ and HCC.^157^ In gastric cancer, the repression of ZNF382 resulted in an increase in tumor cell proliferation, migration, and invasion, and the inhibition of apoptosis. Additionally, ZNF382 silencing had a positive impact on EMT and, thus, on cell metastasis. Furthermore, the *in vitro* data from two gastric cancer cell lines, MKN45, and SGC7901, indicated that ZNF382 repressed the expression of core genes involved in pluripotency maintenance (OCT4, SOX2, and NANOG).^158^

### ZNF331

KRAB-ZFP factors with TSG function also include *ZNF331*, whose promoter methylation occurs in cholangiocarcinoma,^171^ colorectal,^154^ gastric, and pancreatic^172^ cancers. Additionally, methylation of the *ZNF331* promoter was associated with weaker overall survival of colorectal cancer patients.^154^ ZNF331 was shown to have a negative effect on cancer features, as it was demonstrated to reduce proliferation, arrest the cell cycle in the G1/S phase,^154^ and inhibit migratory and invasive properties.^155^ Also, in mouse xenografts from colorectal cancer cells, high expression of ZNF331, compared to gene silencing, resulted in a smaller tumor volume.^154^ Differential proteomics studies suggested that ZNF331 may exert its TSG role through suppression of genes involved in migration and invasion (DSTN, and ACTR3), as well as proliferation (DDX5, EIF5A, GARS, STAM, UQCRFS1, and SET).^155^ Moreover, ZNF331 was demonstrated to reduce the expression of c-Myc oncogene, which is particularly important in terms of CSCs functioning.^173^ Of note, in the case of breast cancer cells, c-Myc overexpression resulted in the acquisition of stemness traits, including the expression of ALDH1 marker.^156^ Unlike in breast cancer cells, a decrease in c-Myc expression due to ZNF331 was reported in gastric cancer.^155^ Further studies are needed to fully understand the role of ZNF331 in different tumors and its role in stemness-related processes.

## Conclusion

KRAB-ZFPs are transcription factors involved in many biological processes. In cancer cells, several members of the family were identified as oncogenes, whereas others act as TSGs.^11^ Still, little is known about their function in CSCs. Certain studies identified the role of specific KRAB-ZFPs in cells with stemness properties, i.e., ESCs or iPSCs. The results shed some light on KRAB-ZFPs' involvement in proliferation, differentiation, and stemness maintenance.^7^^,^^134^ Because physiological stem cells share many features with CSCs, it is possible that similar events are regulated by the same factors also within the cancer subpopulation. CSCs are responsible for tumor metastasis and resistance to chemotherapy.^104^^,^^174^ Understanding the mechanisms responsible for their formation and maintenance in the body may be beneficial for cancer treatment. Unraveling the influence of specific KRAB-ZFPs on CSCs may allow their usage in anti-cancer therapies. However, more research is still needed to further characterize this issue.

## Author contributions

Conceptualization: UO, MM; Visualization: AO; Writing – original draft: AO, MM; Writing – review & editing: UO, MM.

## Conflict of interests

Authors declare no conflict of interest.

## Funding

This work was supported by the 10.13039/501100004442National Science Centre, Poland: 2015/17/B/NZ2/03689 to Urszula Oleksiewicz and 2018/31/D/NZ3/03790 to Marta Machnik.
